# Teen driver system modeling: a tool for policy analysis

**DOI:** 10.1186/s40621-018-0164-9

**Published:** 2018-09-17

**Authors:** Celestin Missikpode, Corinne Peek-Asa, Daniel V. McGehee, James Torner, Wayne Wakeland, Robert Wallace

**Affiliations:** 1 Department of Epidemiology, College of Public Health, University of Iowa, Iowa City, IA USA; 2 Department of Occupational and Environmental Health, College of Public Health, University of Iowa, Iowa City, IA USA; 3 Injury Prevention and Research Center, College of Public Health, University of Iowa, S161 CPHB 105 River Street, Iowa City, IA 52242 USA; 4 University of Iowa Public Policy Center, Iowa city, IA USA; 5 Systems Science Program, Portland State University, Oregon, USA

**Keywords:** Teen, Driving events, Dynamics, Systems, Modeling, Policy analysis

## Abstract

**Background:**

Motor vehicle crashes remain the leading cause of teen deaths in spite of preventive efforts. Prevention strategies could be advanced through new analytic approaches that allow us to better conceptualize the complex processes underlying teen crash risk. This may help policymakers design appropriate interventions and evaluate their impacts.

**Methods:**

System Dynamics methodology was used as a new way of representing factors involved in the underlying process of teen crash risk. Systems dynamics modeling is relatively new to public health analytics and is a promising tool to examine relative influence of multiple interacting factors in predicting a health outcome. Dynamics models use explicit statements about the process being studied and depict how the elements within the system interact; this usually leads to discussion and improved insight. A Teen Driver System Model was developed by following an iterative process where causal hypotheses were translated into systems of differential equations. These equations were then simulated to test whether they can reproduce historical teen driving data. The Teen Driver System Model that we developed was calibrated on 47 newly-licensed teen drivers. These teens were recruited and followed over a period of 5-months. A video recording system was used to gather data on their driving events (elevated g-force, near-crash, and crash events) and miles traveled.

**Results:**

The analysis suggests that natural risky driving improvement curve follows a course of a slow improvement, then a faster improvement, and finally a plateau: that is, an S-shaped decline in driving events. Individual risky driving behavior depends on initial risk and driving exposure. Our analysis also suggests that teen risky driving improvement curve is created endogenously by several feedback mechanisms. A feedback mechanism is a chain of variables interacting with each other in such a way they form a closed path of cause and effect relationships.

**Conclusions:**

Teen risky driving improvement process is created endogenously by several feedback mechanisms. The model proposed in the present article to reflect this improvement process can spark discussion, which may pinpoint to additional processes that can benefit from further empirical research and result in improved insight.

**Electronic supplementary material:**

The online version of this article (10.1186/s40621-018-0164-9) contains supplementary material, which is available to authorized users.

## Background

Adolescents make up an important segment of the U.S. population. There were 41.6 million adolescents in 2010 and this population is expected to reach 42.4 million by 2020, and a record of 50 million by 2040 (U.S. Census Bureau, 2000a; U.S. Census Bureau, 2000b). Protecting young people from health risks is critical for a country’s future because healthy adolescents are an important asset and resource for social and economic development. Despite the tremendous efforts made in recent years towards improving overall health status of adolescents, motor vehicle crashes continue to be the most frequent cause of death for individuals 13–19 years of age (http://webappa.cdc.gov/sasweb/ncipc/leadcaus10_us.html). While teen crash fatality rates have declined in recent years, current crash rates still have a serious human and economic cost. In the US in 2016, 2820 teenagers ages 13–19 died in motor vehicle crashes, representing on average 8 deaths each day (http://www.iihs.org/iihs/topics/t/teenagers/fatalityfacts/teenagers). The financial burdens of motor vehicle crashes in adolescence are large and far-reaching. Although young individuals account for only 14% of the U.S. population, they represent 30% ($19 billion) of the total costs of automobile injuries among males and 28% ($7 billion) among females (http://www.cdc.gov/Motorvehiclesafety/Teen_Drivers/teendrivers_factsheet.html).

Epidemiological profiles of teen crash risk show a rise in crash rates immediately post-licensure with a peak during the second month, after which the rate starts to decline (Lewis-Evans, 2010; Chapman et al., 2014). Researchers have collectively explained the decline in teen crash risk by accumulation of driving experience (Mayhew et al., 2003; McCartt et al., 2003; McKnight A J and McKnight A S, 2003). However, the mechanisms underlying the peak in crash rates during the second month after licensure rather than the first month are poorly understood. Hypotheses to explain the peak in crash rates during the second month have broadly focused on increase in driving exposure (Chapman et al., 2014). This hypothesis was dismissed by a study showing that dangerous driving events representing surrogates for crashes peaked during the second month after licensure rather than the first month after accounting for driving exposure (Missikpode et al., 2018). Therefore, the peak in crash rates during the second month post-licensure rather than the first month remains a puzzle.

Research on crash risk factors among novice teen drivers has been intense, and evidence suggests that a diverse range of factors influence teen crash risk. These include but are not limited to: (i) lack of experience; (ii) individual-level factors such as age, gender, maturation; (iii) behavioral factors such as distraction (e.g., cell phone use while driving, teen passengers), driving over the speed limit; (iv) environmental factors such as road conditions and weather (Ivers et al., 2009; Turner and McClure, 2003; Rhodes and Pivik, 2011; Massie et al., 1995; Zwerling et al., 2005; Kmet and Macarthur, 2006). How do we think about, and analytically grapple with, the potential contribution of all these factors influencing teen crash risk? Although lack of experience is clearly linked to crash involvement among novice teen drivers, driving experience is in turn shaped by the number of crash events. For example, an analysis of a naturalistic driving data using a Bayesian multivariate Poisson lognormal model has shown that drivers involved in more crashes in the past year were less likely to be involved in a crash in the next year (Wu et al., 2014). Similarly, even though risk-taking behaviors are linked to increased crash risk, previous crashes could also be a determinant of tendency of engaging in risk-taking behaviors. Moreover, parents may impose restrictions on access to the car when the teen has been involved in a crash, further slowing the teen’s driving experience. It would be a substantial assumptive stretch to argue that there is a linear relationship, for example, between driving experience and crash risk, and even more of a stretch to argue that any hypothesized parametric relation is consistent over time across factors influencing crash involvement. Thus, there are clear interrelations between crash factors, and it is, therefore, likely that these factors are not easily parameterized. Although regression-based models are helpful at isolating independent relationships between covariates and outcomes while accounting for confounders, they are poorly suited to deal with these complications. These traditional research methods do little to take into account the dynamic and reciprocal relations between some ‘exposures’ and ‘outcomes’, discontinuous relations or changes in the relations between ‘exposures’ and ‘outcomes’ over time.

Systems dynamics modeling is an alternative approach, which examines system influences and their interplay, and is able to handle feedback loops and complex interactions between variables. Unlike the traditional epidemiological methodology which often studies risk factors in isolation, this approach investigates how the different crash factors fit together, interact, and change over time. Instead of ignoring these feedback processes, a system dynamic approach explicitly models these processes and investigates how these feedback mechanisms contribute to the patterns observed. This process-driven approach may provide a theoretical explanation for the significant differences in the distribution of crashes within the young driver population as some teenage drivers have no crashes and others have several (Simons-Morton et al., 2006; Simons-Morton et al., 2011, Li et al., 2011; O’Malley and Johnston, 2003). Thus, viewing adolescent crash risk from a systems perspective may allow one to conceptualize crashes that might seem irrational and disparate within a framework that gives meaning and sense to these crashes.

The primary goal of this study was to develop a conceptual framework of teen risky driving improvement curve using system dynamics methodology. Using this conceptual framework as a guide, we leveraged an understanding of the dynamic process underlying patterns in teen risky driving over time. This framework may be used for teen driving policy analysis.

## Methods

### Data source

A sample of newly-licensed teen drivers and at least one of their parents was recruited from high schools in Iowa City and Des Moines, Iowa and surrounding areas for a randomized controlled trial (can be found online at: https://clinicaltrials.gov/ct2/show/NCT01624597). This study analyzed 47 participants assigned to the control group, who received no driving intervention throughout the five months of their study participation. Teenaged drivers were eligible if they received their Intermediate License during the study period, which in Iowa represents the first opportunity for unsupervised driving. The teens recruited had access to a vehicle for which they were the primary driver. At the time of recruitment, drivers were around the ages of 16 years. Consent was obtained from parents and teens, and the study protocols were reviewed and approved by the University of Iowa Institutional Review Board.

Each participant’s vehicle was equipped with in-vehicle instrumentation developed by Lytx to capture driving events and driving trips. The device was installed a few days before or the day the teen received his/her intermediate license. However, data collection started the day the license was obtained and lasted five months. The device had two cameras and was attached to the windshield behind the rear-view mirror of the vehicle, and recorded video of the interior, forward and rearward of the vehicle. This strategic position of the device allowed to determine whether the driver was a study participant. The video recording system, which continuously buffered video, were triggered to record when acceleration/deceleration or lateral movement exceeded a pre-set threshold of 0.5 g, a force that is noticeable and uncomfortable to the vehicle occupants. All triggered events were automatically downloaded from the device and transferred to the University of Iowa laboratory via a secure wireless connection to Lytx, where videos were reviewed to exclude non-participating drivers and false triggers. All eligible triggered events were reviewed and reconciled by two trained coders. All videos were reviewed to check for any risky driving behaviors (e.g., driving over the speed limit, cell phone use while driving). Data were also collected on miles traveled through odometer readings of the video recording system.

For this study, the main outcome of interest was driving events. Driving events spanned the full range of g-force events, near-crash events, and crash events. G-force events were detected by accelerometers on the video recording system, and occurred as a result of accelerating rapidly, decelerating late and abruptly, sharp cornering, and over-correcting after a turn. A g-force event occurred when the driver’s actions resulted in a g-force > 0.5 g or g-force <− 0.5 g. A study has shown that g-force events are useful surrogates for crashes (Simons-Morton et al., 2012). Near-crash events were g-force events in which an evasive maneuver was performed by the teenager in order to avoid a collision (braking > 0.65 g, deceleration > 0.5 g in < 0.5 s; proximity to another vehicle/object; squealing of tires). Crash events were events that resulted in a collision with an object or another vehicle. Events that were not the teenage driver’s fault (e.g., braking for another vehicle) were not included in the current analyses.

### Model development

**Fig. 1:**
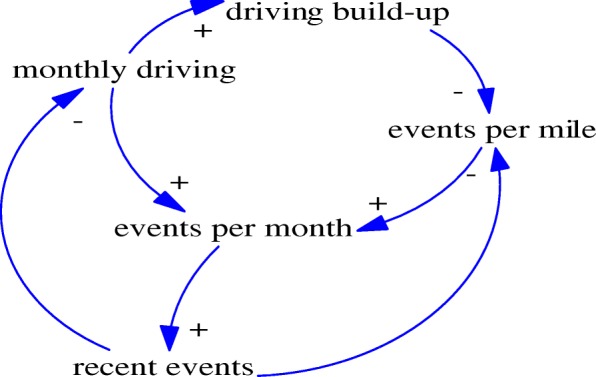
Causal loop diagram. The single arrows (in blue) represent causal relationships. A causal relationship linking together two variables is represented by an arrow pointing from the independent to the dependent variable. A sign (+) means the independent variable causes the dependent variable to increase. A sign (−) means the independent variable causes the dependent variable to decrease

Dynamics models use explicit statements about system structure to map a problem and depict how the elements within the system interact (Sterman, 2000). Figure 1 presents the causal diagram resulting from our work to conceptualize factors shaping teen crash risk. We developed this conceptual framework via an iterative process. Key hypothesized factors influencing the dynamics of teen crash risk include accumulated driving and recent crashes. Accumulated driving (i.e., accumulated mileage at the wheel) causes teen crash risk to decline over time (Graham and Gootman, 2008), as it allows the novice driver to develop the skills and attitudes essential for safe driving. Recent crashes are associated with decreased crash risk in the future (Wu et al., 2014). The underlying mechanisms of this association are not known. This association may be partially mediated via risk-taking behaviors (e.g., driving over the speed limit) as recent crashes may decrease the tendency of engaging in risk-taking behaviors. While acknowledging the importance of risk-taking behaviors as an important crash risk factor, our model followed the assumption that this variable is an intermediate variable between recent crashes and current crash risk. The model further assumes that recent crashes are associated with less driving, perhaps via fear of driving or driving restrictions imposed by parents because of crash involvement. For this study, we combined crash events and their surrogates (g-force and near-crash events) into a single measure called “events”. Our dynamic hypothesis is that the natural teen risky driving improvement curve is slow improvement followed by faster improvement, and finally a plateau: that is, an S-shaped decline in driving events.

**Fig. 2:**
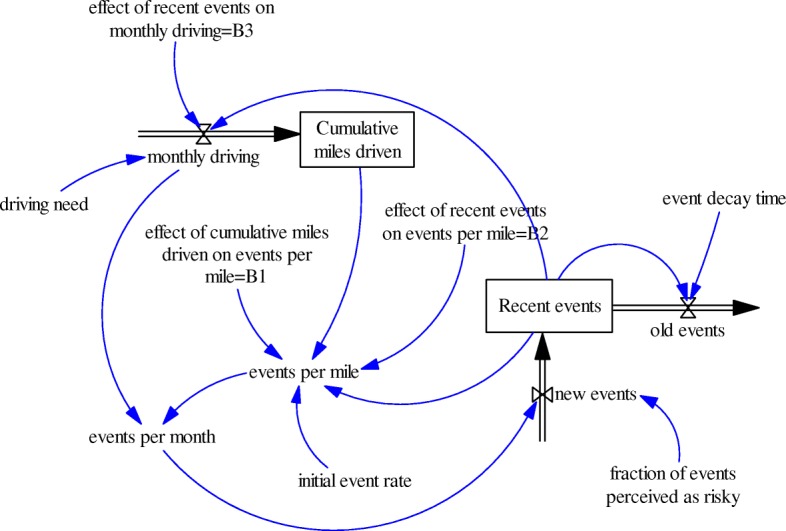
Teen Driver System Model. The single arrows (in blue) represent causal relationships. The rectangles (or boxes) represent stocks (which accumulate flows over time). The double arrows with a valve symbol represent flows. A double arrow going in a stock is called an inflow and a double arrow going out of a stock is called an outflow. Inflows increase the value of a stock whereas outflows decrease the value of a stock

Through an extensive process of testing and evaluation, several model structures were investigated. The model presented in Fig. 2 emerged from that process as the simplest dynamic hypothesis that adequately captured patterns in teen risky driving. This model is a deterministic ordinary differential equation model that captures the interactions among amount of driving, events per month, recent events, events per mile, and their evolution through time. The model was developed to reproduce monthly amount of driving and monthly driving events for each teenage driver, while also seeking to understand the dynamic processes underlying these time series. The results of two other simple models that were considered but failed model validity tests can be found in the Additional file 1. These models failed behavioral tests, that is, the dynamic patterns generated by these models were not consistent with the historical teen driving data patterns.

### Model description

#### Model structure

Figure 2 presents the system structure diagram resulting from our work to conceptualize teen risky driving improvement curve. The building blocks of any system dynamics model are feedback loops. A feedback loop is a chain of variables interacting with each other in such a way they form a closed path of cause and effect relationships. Variables that are part of loops are called endogenous variables whereas variables that are not part loops are called exogenous or external variables. The values of endogenous variables are calculated with every time-step of the system dynamics model and therefore they change in time. Endogenous variables which accumulate over time are referred to as stocks and are represented by rectangles in system dynamics diagrams. Stocks are also known as state variables and represent the state of the system at each time point. “Cumulative miles driven” and “recent events” are the stock variables in the Teen Driver System Model. Endogenous variables governing the levels of stock variables are referred to as flows and depicted as a double arrow representing the direction of the flow and a valve symbol representing the fact that the flow quantity is being regulated. Flows which increase the value of a stock are called inflows, whereas those which decrease it are called outflows. Monthly driving is an inflow as it causes “Cumulative miles driven” to increase. The variable “new events” is an inflow as it causes “recent events” to increase whereas the variable “old events” is an outflow as it causes “recent events” to decrease.

Exogenous variables can have a predefined pattern or a constant value. If external variables have a constant value, they are referred to as constants or parameters. Driving need, initial event rate, fraction of events perceived as risky, event decay time, effect of recent events on monthly driving, effect of recent events on events per mile, and effect of cumulative miles driven on events per mile are model constants. The inflow of “monthly driving” is regulated by the stock variable “recent events”, which has a negative feedback on monthly driving. Driving need is the amount of driving when the value of recent events is equal to zero. The inflow of “new events” depends on the variable “events per month” and the fraction of events perceived as risky. The outflow of “old events” depends on “recent events” and event decay time. The variable “event decay time” is a conceptual factor and is defined as the average time for an event to become old. The variable “events per mile” is calculated based on the stocks “cumulative miles driven” and “recent events”, and both have a negative effect on events per mile. The variable “initial event rate” is events per mile (the baseline event rate or initial risk) when both recent events and cumulative miles driven are equal to zero. Initial event rate may be a function of fixed variables such as age, gender, and maturity level. For example, a study conducted by Chapman and colleagues on trends in crash rates across different age groups showed that the higher the age, the lower the baseline crash rates (Chapman et al., 2014). The variable “fraction of events perceived as risky” represents the proportion of events perceived by the teen as risky. We assumed that only collisions are perceived as risky and they represented on average 7% of all teen driving events. We further assumed that environmental factors (e.g., road and weather conditions) are exogenous variables in the model.

The model was built in Vensim, which is a simulation software program used for developing and analyzing system dynamic models (http://vensim.com/vensim-software/).

### Calibration of model parameters

We denoted the effect of cumulative miles driven on event per mile as β1; the effect of recent events on event per mile as β2; and effect of recent events on monthly driving as β3. These assumptions produced the set of ordinary differential equations for an individual i.

The differential equation of the stock “recent events” is as follows:

$$ \frac{drecent\ events}{dt}= new\  events-\frac{recent\ events}{event\ decay\ time} $$

Since the measurements are frequent in comparison with the delays involved in the balancing feedback loops, the equation can be converted to a difference equation:

$$ \frac{\Delta recent\ events}{\Delta t}= new\ {event s}_{t-1}-\frac{{recent\ events}_{t-1}}{event\ decay\ time} $$

Recent events(t) = Recent events(t-1) + dt(new events(t)-old events(t)).

The differential equation of the stock “cumulative miles driven” is as follows:

$$ \frac{dcumulative\ miles\ driven}{dt}= monthly\ driving $$

The equation can be converted to a difference equation:

$$ \frac{\Delta \mathrm{cumulative}\ \mathrm{miles}\ \mathrm{driven}}{\Delta t}={monthly\ driving}_{t-1} $$

Cumulative miles driven(t) = cumulative miles driven(t-1) + dt(monthly driving(t)).

Event per mile at time t is determined as:

$$ {\displaystyle \begin{array}{l}\mathrm{Event}\ \mathrm{per}\ \mathrm{mile}\left(\mathrm{t}\right)=\exp \left(\upbeta 0+\upbeta {1}^{\ast}\mathrm{cumulative}\ \mathrm{miles}\ \mathrm{driven}\left(\mathrm{t}\right)+\upbeta {2}^{\ast}\mathrm{recent}\ \mathrm{event}\mathrm{s}\left(\mathrm{t}\right)\right)\\ {}\mathrm{Event}\ \mathrm{per}\ \mathrm{mile}\left(\mathrm{t}\right)=\exp \left(\upbeta 0\right)\ast \exp \left(\upbeta {1}^{\ast}\mathrm{cumulative}\ \mathrm{miles}\ \mathrm{driven}\left(\mathrm{t}\right)+\upbeta {2}^{\ast}\mathrm{recent}\ \mathrm{event}\mathrm{s}\left(\mathrm{t}\right)\right)\\ {}\mathrm{Event}\ \mathrm{per}\ \mathrm{mile}\left(\mathrm{t}\right)=\mathrm{initial}\ \mathrm{event}\ {\mathrm{rate}}^{\ast}\exp \left(\upbeta {1}^{\ast}\mathrm{cumulative}\ \mathrm{miles}\ \mathrm{driven}\left(\mathrm{t}\right)+\upbeta {2}^{\ast}\mathrm{recent}\ \mathrm{event}\mathrm{s}\left(\mathrm{t}\right)\right)\ \mathrm{where}\ \exp \left(\upbeta 0\right)=\mathrm{initial}\ \mathrm{event}\ \mathrm{rate}\end{array}} $$

Monthly driving at time t can be expressed as:

$$ {\displaystyle \begin{array}{l}\mathrm{Monthly}\ \mathrm{driving}\ \left(\mathrm{t}\right)=\upbeta 0+\upbeta {3}^{\ast}\mathrm{recent}\ \mathrm{events}\left(\mathrm{t}\right)\\ {}\mathrm{Monthly}\ \mathrm{driving}\ \left(\mathrm{t}\right)=\mathrm{driving}\ \mathrm{need}+\upbeta {3}^{\ast}\mathrm{recent}\ \mathrm{events}\left(\mathrm{t}\right)\ \mathrm{where}\ \upbeta 0=\mathrm{driving}\ \mathrm{need}.\end{array}} $$

Model parameters were estimated using calibration, which involves finding values of unknown parameters to best match historical data. We performed the model validation on both the aggregate historical data and individual level historical data. We set some parameters as fixed effects (parameters with the same value across all teens) because we thought these parameters should not be very different across subjects. These parameters were event decay time, effect of recent events on monthly driving, effect of recent events on events per mile, and effect of cumulative miles driven on events per mile. An internal optimization engine of the simulation software, which utilizes a modified Powell gradient search method, was used to find the parameters that minimized mean squared error. To ensure convergence for this nonlinear optimization, we conducted 648,265 independent searches from random points on the parameters pace. In this paper, we report both the actual and predicted data for the calibration of the aggregate data. Actual and predicted data at driver level are provided in Additional file 1. The predicted data integrates historical data and predicts into the future. The model fit provides a measure of the model quality for the mechanisms operating in the system.

Theil inequality statistics provide the summary statistics for historical fit (Sterman, 1984) and are a standard validity test for system dynamics models. The Theil statistics decompose model-data variance into three terms: difference in means Um, difference in variance Us, and difference in covariance Uc. Um is the fraction of Mean Squared Error (MSE) due to difference in means (fraction of the MSE due to bias). A large Um indicates systematic bias, reflecting a difference between the model and reality. Us is the fraction of Mean Squared Error (MSE) due to difference in variance. Uc is the fraction of Mean Squared Error (MSE) due to point-to-point covariance. These summary statistics (Um, Us, and Uc) sum to 1. Other performance metrics are the total Root Mean Squared Error (RMSE) between model and data, and the Mean Absolute Percentage Error (MAPE), which is the sum of ABS((data-model)/data) multiplied by 100.

## Results

### Historical data trends

**Fig. 3:**
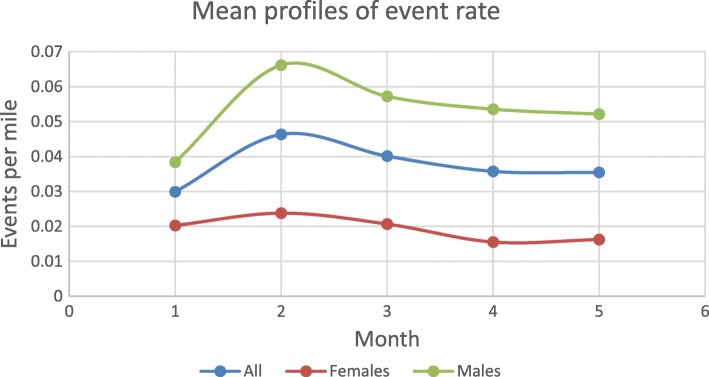
Mean event rate profiles for the study population and for males and females. The blue curve represents the trend in mean event rate (events/mile) for the study population. The red curve represents the trend in mean event rate (events/mile) for females. The green curve represents the trend in mean event rate (events/mile) for males

Table 1 shows means of events, miles driven, and event rate over time. There was an increase in the means of events and event rate over the first two months, after which there was a decline. The monthly miles traveled appeared to be increasing over time. Figure 3 shows the mean event rate profiles for the study population  and for males and females. For both males and females, there is a peak in event rate during the second month followed by a slow decline.

**Table 1:** Distribution of events, miles driven, events per mile over time

Month	Events Mean (SE)	Miles driven Mean (SE)	Events per mile Mean (SE)
1	18.91 (37.50)	548.85 (242.87)	0.03 (0.05)
2	26.57 (49.68)	580.85 (287.32)	0.05 (0.09)
3	21.11 (34.89)	581.64 (303.39)	0.04 (0.07)
4	18.17 (33.07)	559.53 (303.73)	0.03 (0.06)
5	19.63 (36.25)	629.60 (316.11)	0.03 (0.07)

### Model fit

**Fig. 4:**
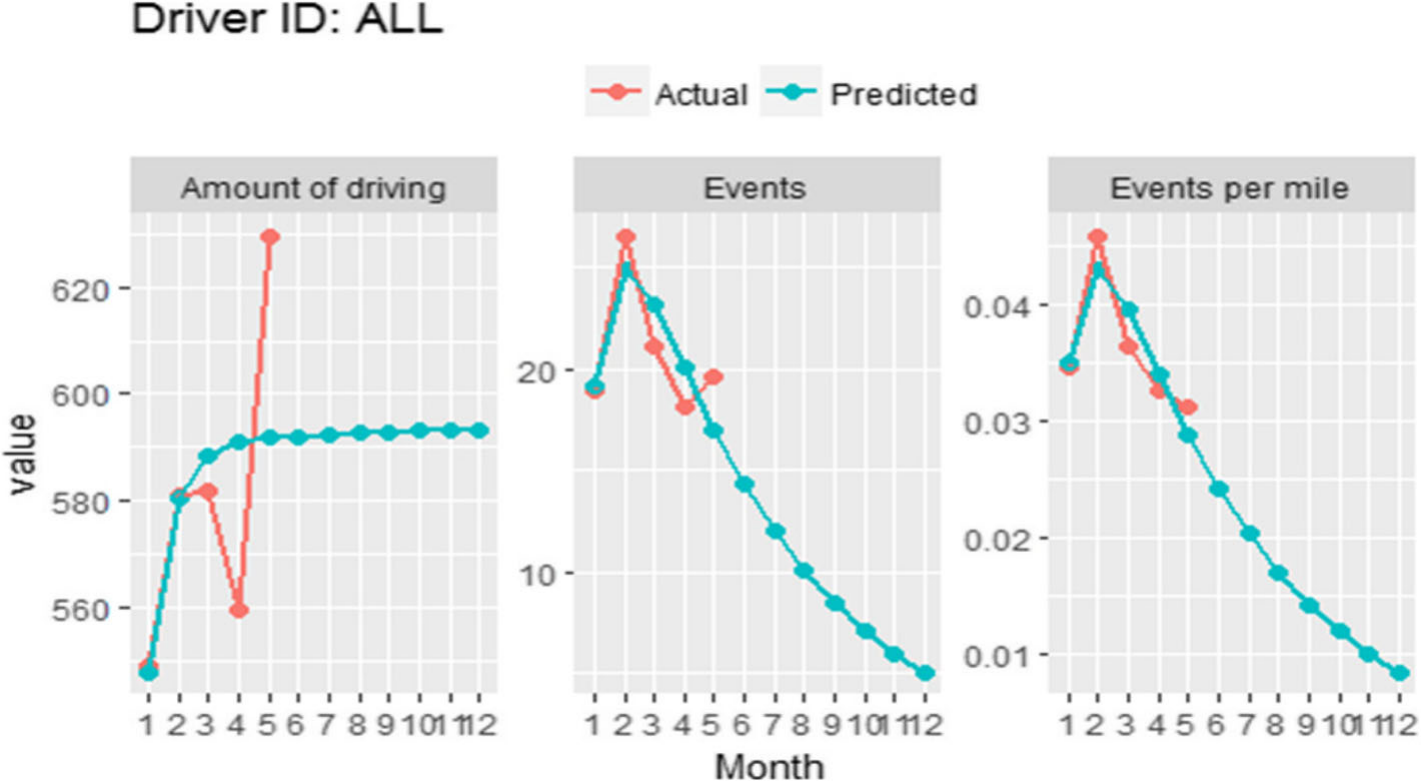
Sample historic trends in driving data and calibrated model simulations. The red curve represents the actual (mean) driving data of the study population and the blue curve represents the predicted driving data

We tested the model using two different levels of data. First, we tested the model using the aggregate data (average events and average amount of driving). To save space, we reported the calibration results of this first validation test (Fig. 4). Second, we tested the model against 47 individual data series. We have provided the graphs of the 47 teens in Additional file 1. The overall behavior (both for the aggregate data trends and individual level data trends) appears to fit the empirical trends. The estimated parameters from the model are listed in Table 2. Theses parameters were optimized using data of the 47 teen drivers. As hypothesized, the model calibration shows that cumulative miles driven has a negative effect on event per mile, and recent events have a negative effect on both event per mile and monthly amount of driving. This confirms the negative feedback exerted by past events on future events and amount of driving. Individualized trends in monthly driving events were influenced by initial event rate (initial risk) and driving need.

**Table 2:** Parameter values for the dynamic model

Parameters	Estimated values and 95% CI
Effect of cumulative miles on event per mile (β1)	−0.00025 (− 0.00027, − 0.00023)
Effect of recent events on event per mile (β2)	−0.00800 (− 0.00827, − 0.00758)
Effect of recent events on monthly driving (β3)	−3.40216 (− 3.59304, − 3.27073)
Event decay time (in month)	6.15949 (5.68868, 6.69635)

We observed significant variations in the model fit across different teen drivers. Thus, there were some individual differences in teen driving behavior that can be taken into account by adding an individualized input as an exogenous variable to capture these individual differences that were not observable, such as daily routines, parent imposed driving restrictions, mechanical auto problems.

#### Model performance metrics

The model performance metrics are shown in Table 3. For the variable “events/month”, the inspection of the fit statistics suggests that only 6% of the mean squared error are due to systematic bias, but the majority of the error is due to differences in variance (56%) and covariance (37%) between data and simulations. The largest fraction of error attributable to unequal variances could potentially be explained by individual differences not captured by the model. The error decomposition for the variable “driving/month” shows that only 5% of the mean squared error is due to systematic bias but the largest fraction of error comes from covariance (63%) and variance (32%). The fact that the error is driven by unequal covariation indicates that simulated monthly driving tracks the underlying trend in the actual monthly driving almost perfectly, but diverges point-by-point. This divergence may be due to noise in the historical data not captured by the model.

**Table 3:** Summary statistics for historical fit

Variable	RMSE	Theil_Um	Theil_Us	Theil_Uc	MAPE
Events/month	7.37 (11.21)	0.06 (0.24)	0.56 (0.30)	0.37 (0.28)	64.43 (48.56)
Driving/month	122.67 (80.53)	0.05 (0.07)	0.32 (0.31)	0.63 (0.33)	21.09 (14.22)

*RMSE* the total Root Mean Squared Error, *MAPE* mean absolute percentage error

## Discussion

We found that teen risky driving improvement process is created endogenously by several feedback mechanisms. The analysis suggests the existence of one reinforcing loop and three negative feedbacks in the model. The reinforcing loop arises from a decline in recent events leading to a faster increase in monthly amount of driving. The increase in monthly amount of driving leads to a faster buildup of miles driven and thus a greater decrease in driving event  rate. The decrease in driving event  rate leads to a further decline in recent events via a slow replenishing of the stock “recent events.” The first negative feedback is between recent events and a resultant decline in the amount of driving. Factors such as fear of driving or imposed restrictions on access to the car could be the mechanisms for this negative feedback, although these variables were not measured and therefore could not be included in the model. The decline in amount of driving would slow down accumulation of driving experience. Reflecting on the behavior of recent events, we would expect that the amount of monthly driving will eventually begin to stabilize as recent events begin to approach zero. By this negative feedback mechanism, more recent events lead to less driving; and less recent events result in more driving. The second negative feedback in the model is a greater number of recent events leading to a greater decrease in event rate, perhaps via corrective actions taken by the teen driver. Thus, more recent events in stock lead to a decrease in event rate, but slow accumulation of amount of driving via less driving. The third negative feedback is controlled by outflow (old events) from the stock of recent events and this outflow is regulated by event decay time. Taken together, the results suggest that variations of individualized trends in driving outcomes are more likely due to significant variations in the stock of “cumulative miles driven”, the stock of “recent events”, and the initial risk (initial event rate).

Consistent with other studies, we found that driving events peak during the second month after licensure rather than the first month (Lewis-Evans, 2010; Chapman et al., 2014). Our methodological approach provides an explanation for this phenomenon, which was not fully understood, overlooked, or dismissed in favor of hypotheses of increased driving exposure. The results presented here provide evidence that such observed trends in driving events have resulted from a simpler set of epidemiological processes. The amount of driving starts moderate and the initial risk (i.e., initial event rate) starts moderate. This leads to more events, which in turn lead to more corrective actions taken by the teen to avoid events (i.e. more caution) but less amount of driving (perhaps via fear or restrictions on access to the car); gradual build-up of experience and slow decline in events; this leads to gradual increase in amount of driving, faster increase in experience, and so a faster decline in events, and finally a plateau. That is a S-shaped decline in driving events.

This model could have important policy implications. The teen driver model presented here is continuously calculating all the model variables and their interactions as part of simulated driving performance. Mathematically, this differential equation model has two important effects. One is to preserve the continuous nature of variables affecting risky driving. For example, the variable “cumulative miles driven” of every teen driver is calculated continuously as part of a teen’s driving performance and can be measured at any instant. All the variables affecting risky driving and their interactions are calculated in this way. In addition, using differential equations makes the model completely continuous in time, allowing any event to occur at any time.

This complex interplay of the timing of driving events and accrual of driving experience is important because many teen driving interventions have options for implementation timing. For example, some interventions may be most effective if implemented during the learner period, while some may be more effective if implemented at the start of unsupervised driving. Furthermore, the fact that the model is continuous in time provides options to explore differential impact based on timing of implementation, assisting decisions on when to initiate, modify, or switch off a possible intervention strategy for a particular teen driver. The model can be used to evaluate the effectiveness of an intervention at a particular time. The model can also be asked to reproduce or predict the results of real clinical trials. The calibrated model presented here offers value as a tool for examining how trends in teen risky driving might behave under a variety of intervention scenarios. For example, the model can be extended to explore the dynamics of introducing teen driving interventions or policies (e.g., driver feedback intervention) and assess intervention tradeoffs.

This study may have methodological implications as related to teen driving research. We found that there is an endogenous process underlying teen risky driving. Teen risky driving results from complex interrelated processes such as self-reinforcing and self-correcting feedback mechanisms. Regression-based approaches used in public health are ill-equipped to investigate problems embedded in feedback processes. Despite their strengths in accounting for potential confounding factors, a major limitation of traditional public health analyses, which are usually regression-based, lies within their inability to account for feedback mechanisms (Luke and Stamatakis 2012; Galea and Ahern 2006). Ignoring these phenomena can limit the ability of statistical models to identify exposure-outcome relationships. For example, a system-based approach was in a used in an epidemiologic study to capture relationships not identified by statistical regression approaches (Auchincloss and Diez Roux, 2008). Studies have reported less predictive performance and reliability for statistical models in the absence of accounting for feedback processes (Lyneis, 2000; Liehr et al., 2001; Srijariya et al., 2008).

Although we have based the model on the best available information pertaining to teen risky driving, one limitation of is the inability of a video recording system to perfectly measure driving performance. The in-vehicle system measures g-force changes, and not all driving errors result in changing g-forces. Additionally, risk-taking behaviors such as running stop signs and cell phone use, as well as parent engagement in their teens’ driving, were not continually measured with this in-vehicle system approach. It is possible that these variables are a part of the endogenous process, or they may be exogenous variables in the model. Despite these uncertainties, the model presented here did match real data, building confidence that the model is “realistic”, even if there are gaps in the current understanding of the dynamics of teen risky driving. As teen risk-taking behaviors and parent engagement become better understood, they can be correctly specified into the model. A very important feature of the model as formulated is that it is easy to expand and update.

Another limitation with the model pertains to selection bias. The model was calibrated using data from a clinical trial, and clinical trials are subject to both random and systematic bias. The main source of systematic bias in this study is selection bias, as participants were volunteers and were not a random sample of the teen driver population. This could be a source of uncertainty in the model parameters.

Another potential limitation of this study is the sample size, which was small, and the relatively short follow-up period. However, our main goal was to make explicit our hypothesis about the dynamics of teen driving behavior, hoping to inspire further discussion and much needed research to further clarify certain relationships. Throughout this investigation, the limited data contributed to adding confidence to the model assumptions and structure. This is an early work, and we hope to refine the model based on critique and suggestions made by the scientific community. After refinement, the model would need to be recalibrated on a much larger study over a longer follow-up period.

It is our hope that the dynamic model presented here will spark further discussion and research on teen driving. We present conclusions from this early work in the hope that some suggestions will resonate with teen driving researchers. While the systems approach used in this study may aid in systematically thinking through innovative teen driving interventions, we believe that our explicit dynamic hypotheses raise opportunities for community reflection, critique, and refinement. The model will be made widely available over the website of the University of Iowa Injury Prevention Research Center, through a very user-friendly interface for free. This will enable the scientific community to interact with the model, view the assumptions and equations, explore how the model functions, and share their comments.

## Conclusions

Teen risky driving follows a course of a slow improvement, then a faster improvement, and finally a plateau: that is, an S-shaped decline in driving events. This improvement process is created endogenously by several feedback mechanisms. The model proposed in the present article to reflect this improvement process can spark discussion, which may result in improved insight. The model may help policymakers design appropriate interventions and evaluate their impacts.

## Additional file

Additional file 1: Fit of the presented model across 47 teen drivers as well as other model structures examined. (DOCX 764 kb)
