# Supplementary material for: Teen driver system modeling: a tool for policy analysis
Source: Inj Epidemiol. 2018 Sep 17;5:34. doi: 10.1186/s40621-018-0164-9 (PMC6139293; doi:10.1186/s40621-018-0164-9)

**Additional file 1**

**Calibration of the Dynamic Model without cumulative miles driven as a stock**

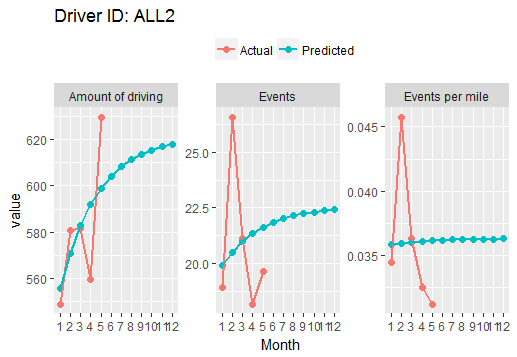


**Calibration of the Dynamic Model without recent events as a stock**

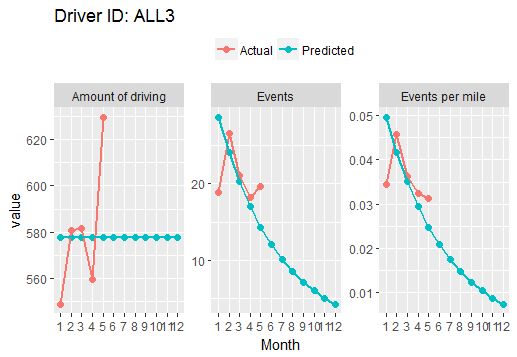


Individual historic trends in driving data and calibrated model simulations


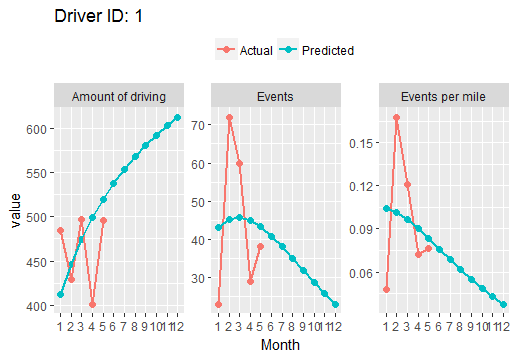


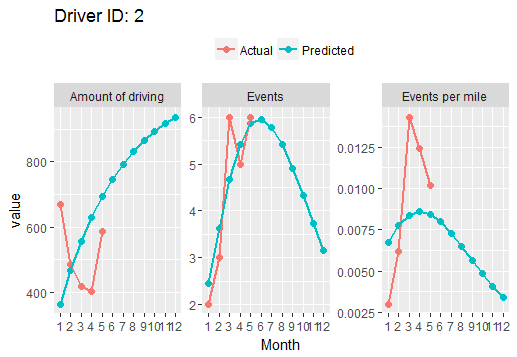


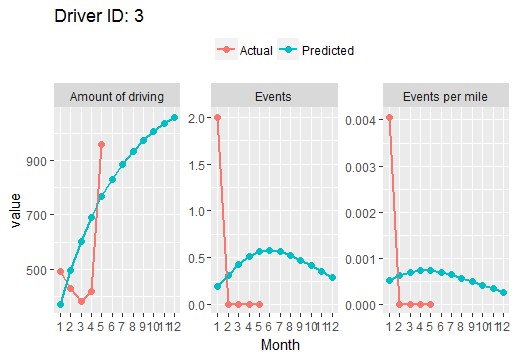


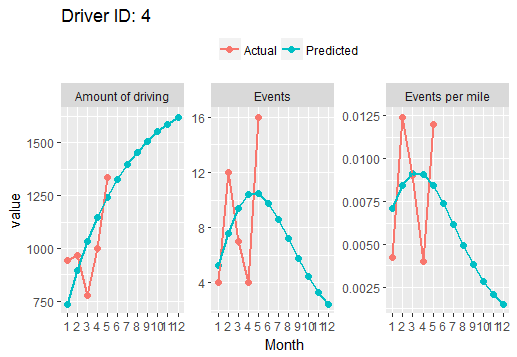


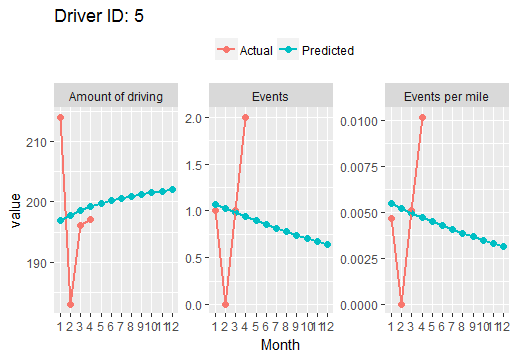


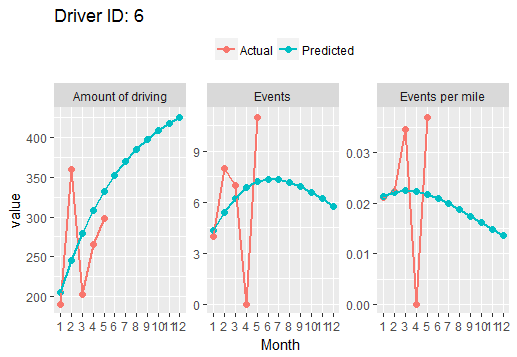


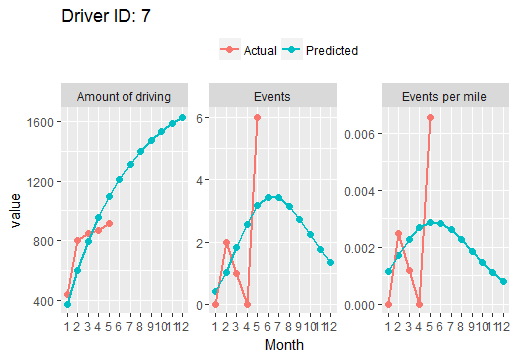


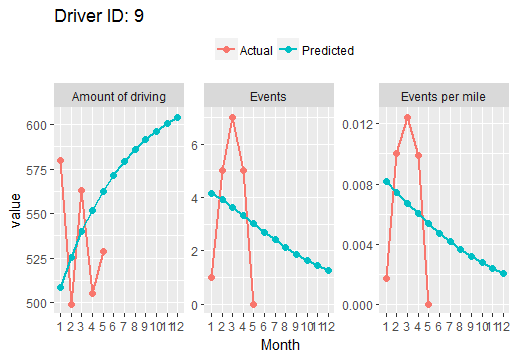


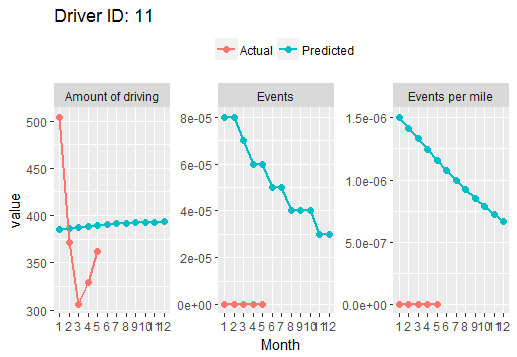


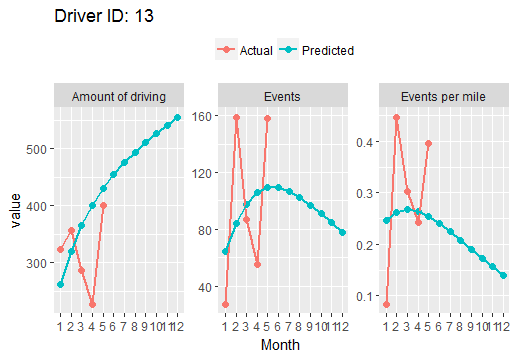


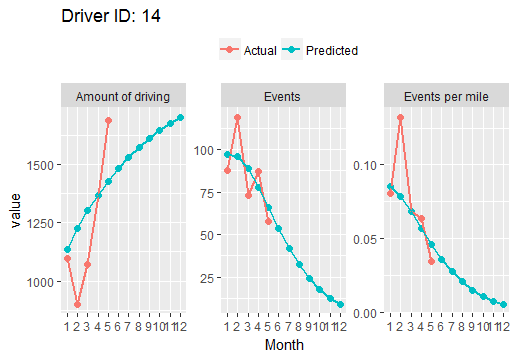


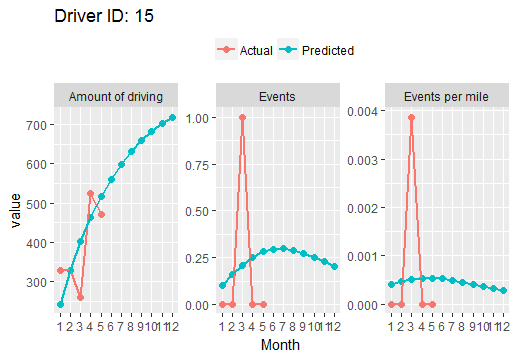


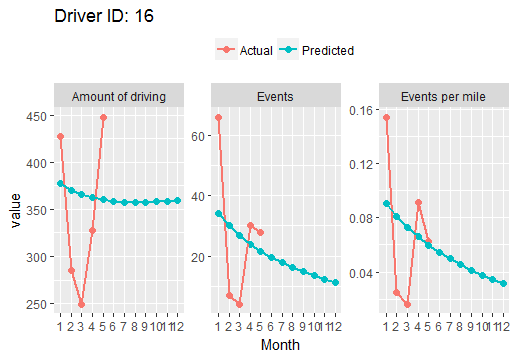


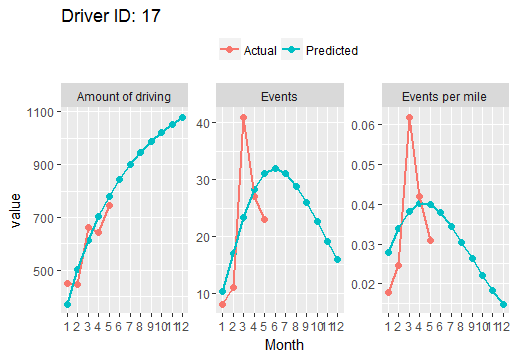


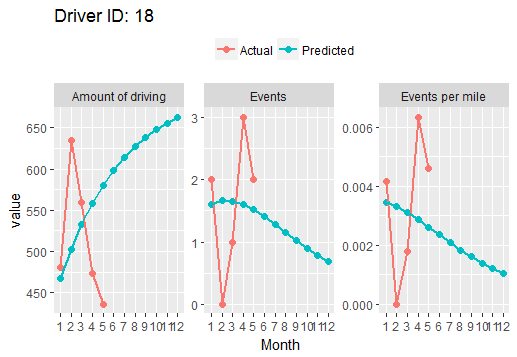


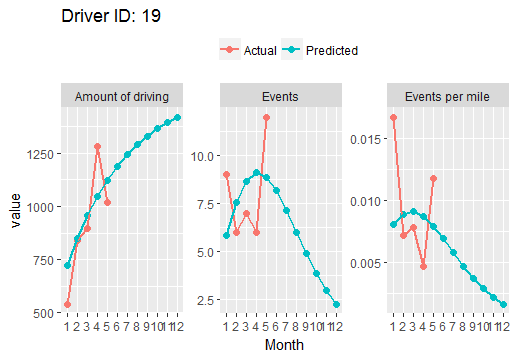


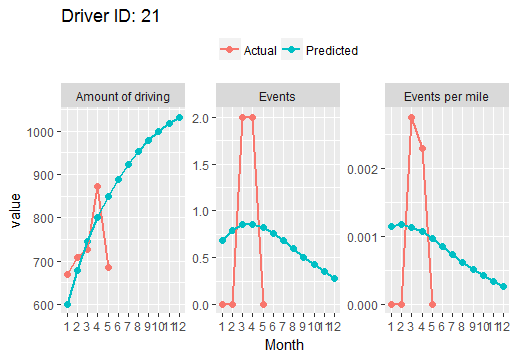


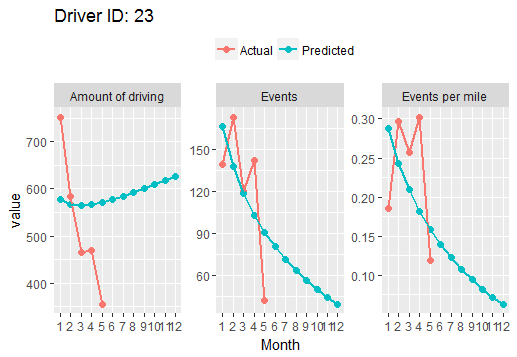


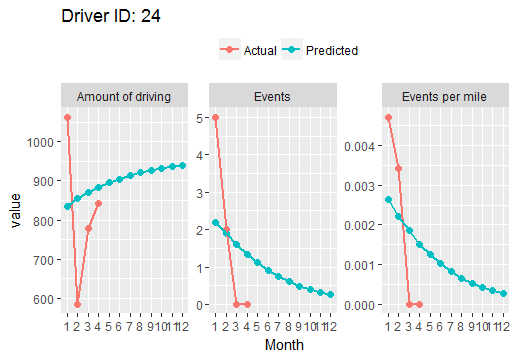


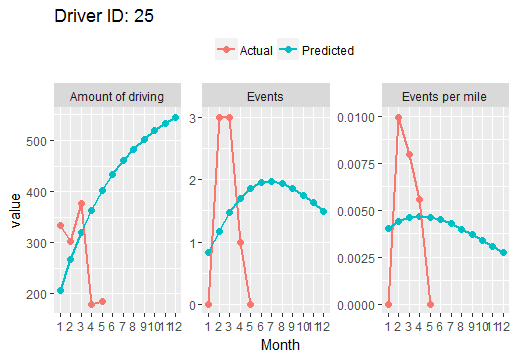


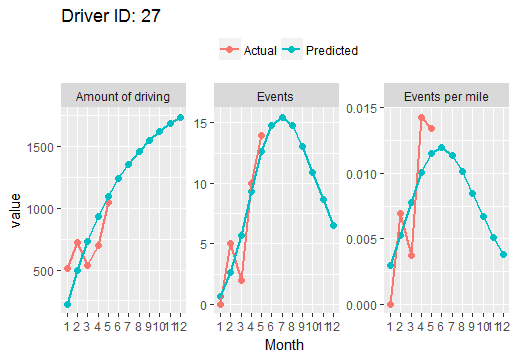


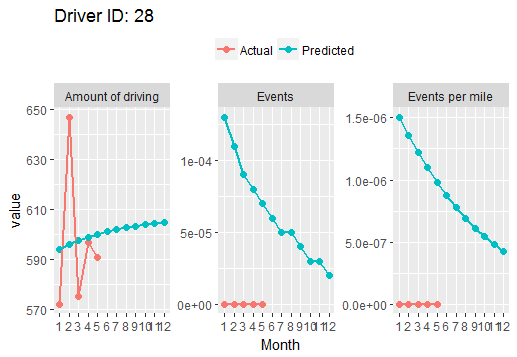


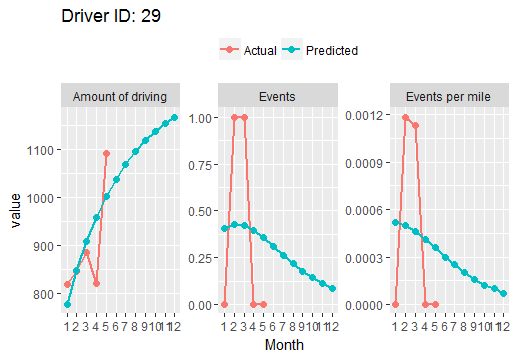


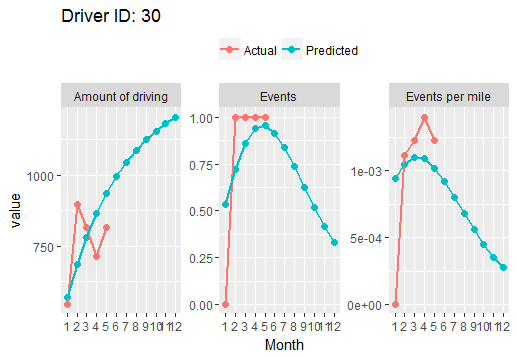


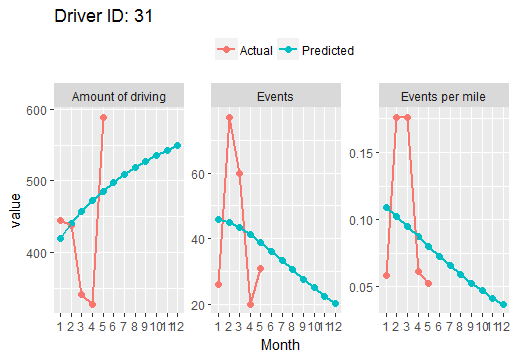


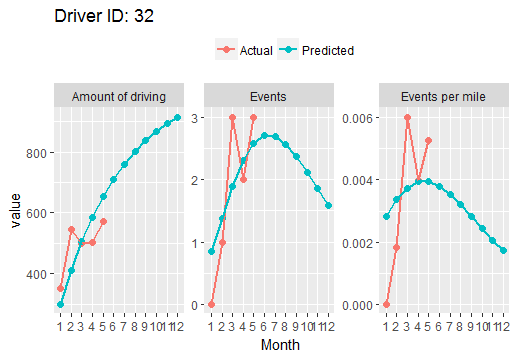


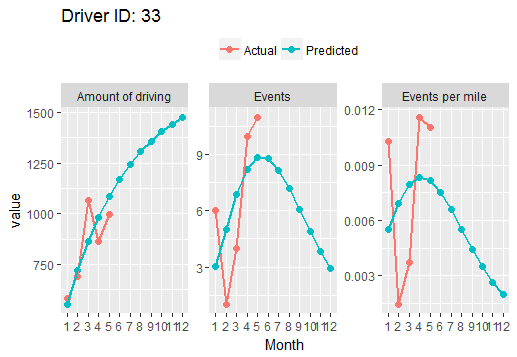


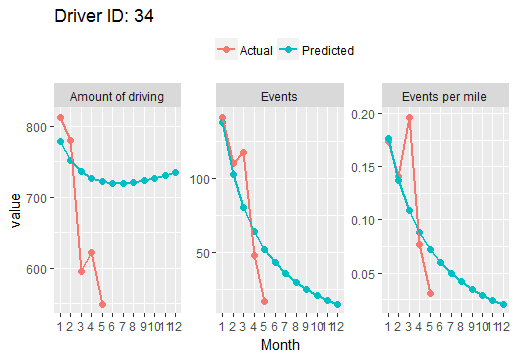


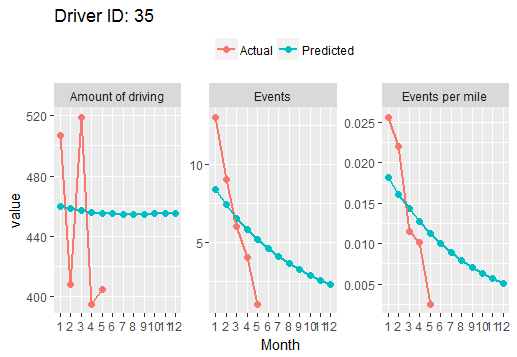


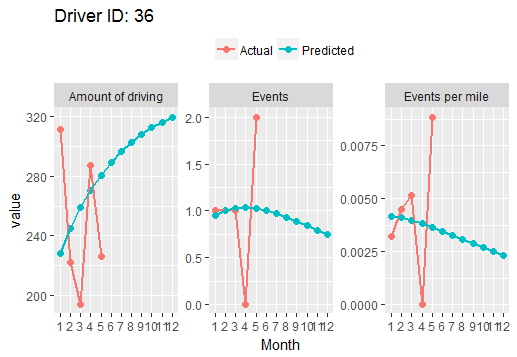


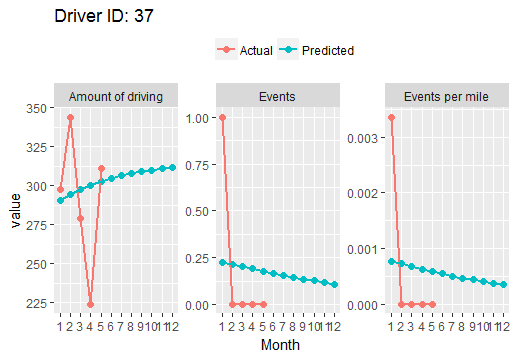


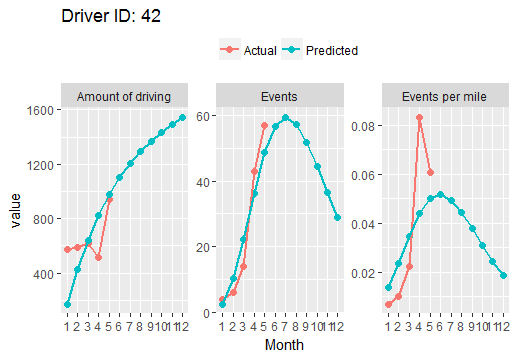


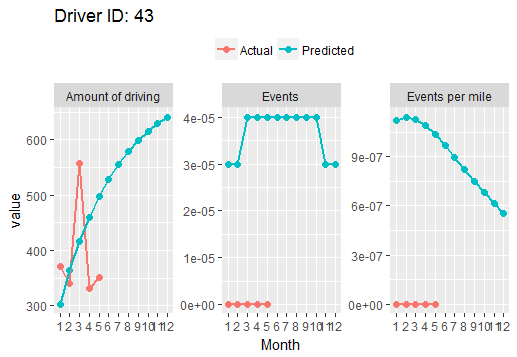


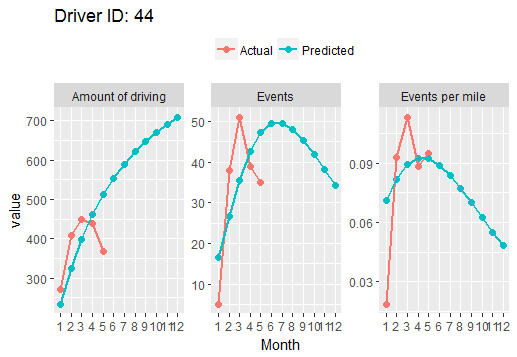


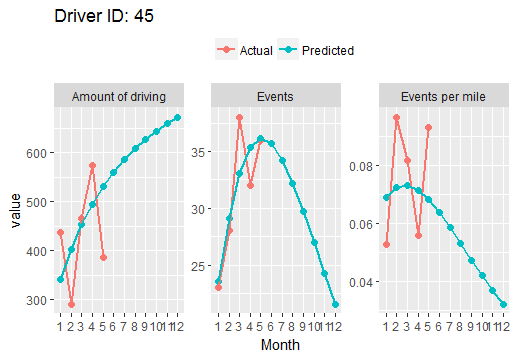


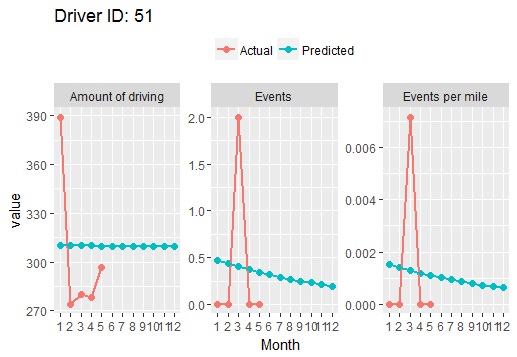


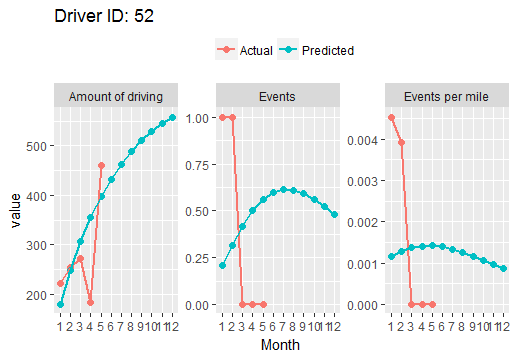


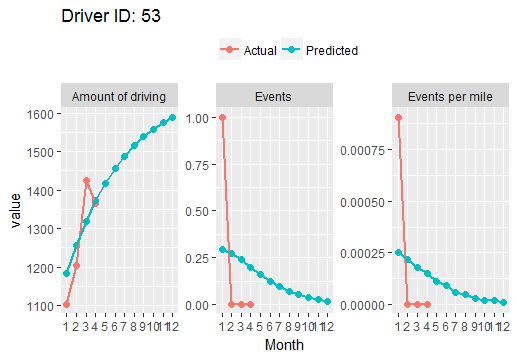


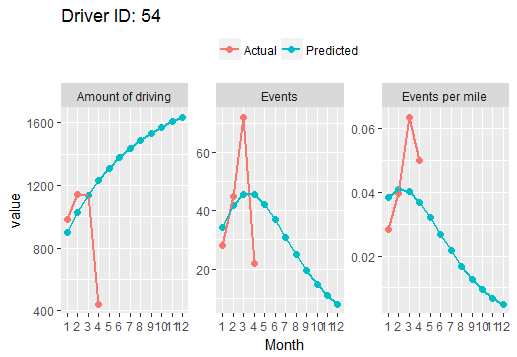


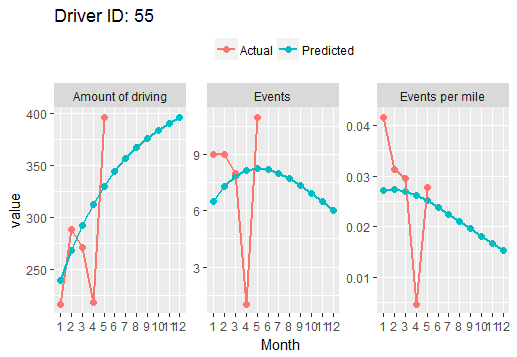


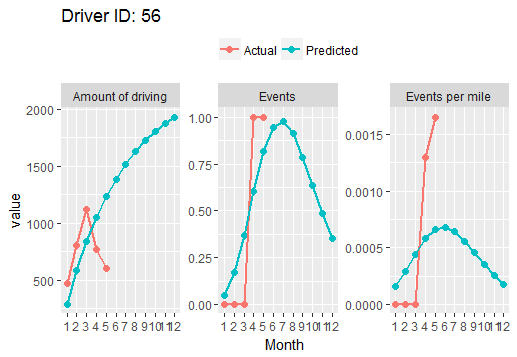


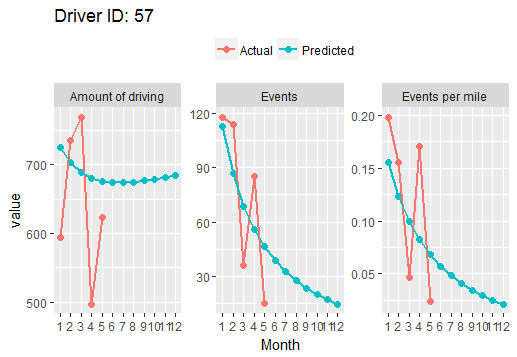


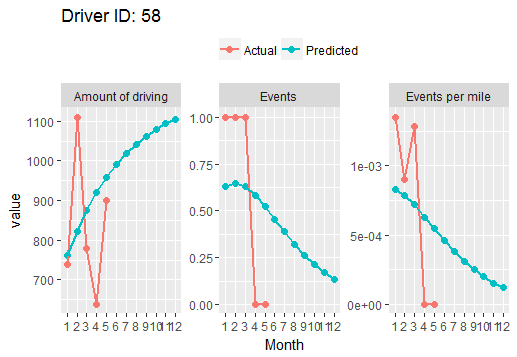


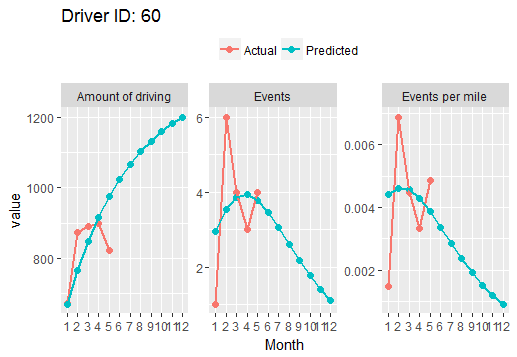


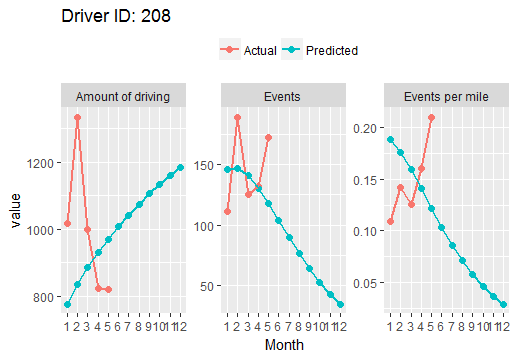


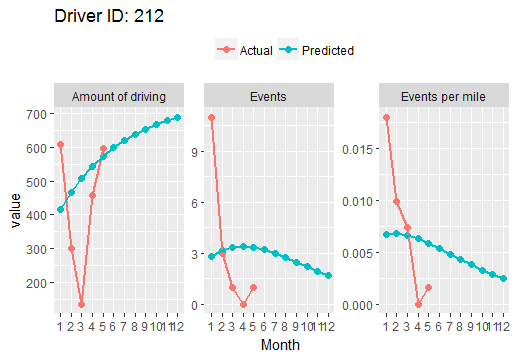


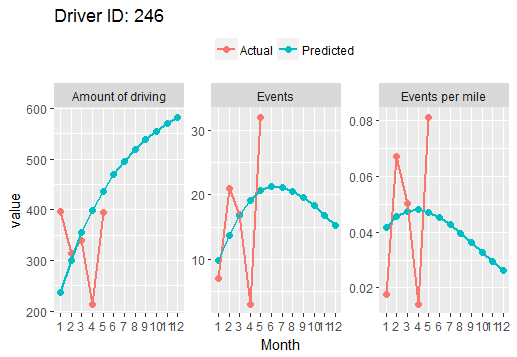

Supplement: Supplementary file 1 — Fit of the presented model across 47 teen drivers as well as other model structures examined. (DOCX 764 kb) [file 40621_2018_164_MOESM1_ESM.docx]
